# Comparative Pharmacokinetics of Gallic Acid After Oral Administration of Gallic Acid Monohydrate in Normal and Isoproterenol-Induced Myocardial Infarcted Rats

**DOI:** 10.3389/fphar.2018.00328

**Published:** 2018-04-06

**Authors:** Zhe Yu, Fan Song, Yu-Chen Jin, Wei-Min Zhang, Ya Zhang, En-Jun Liu, Dan Zhou, Lin-Lin Bi, Qian Yang, Hua Li, Bang-Le Zhang, Si-Wang Wang

**Affiliations:** ^1^Department of Pharmaceutical Analysis, School of Pharmacy, Fourth Military Medical University, Xi’an, China; ^2^Department of Natural Medicine, School of Pharmacy, Fourth Military Medical University, Xi’an, China; ^3^Cadet Brigade, Fourth Military Medical University, Xi’an, China; ^4^Department of Pharmaceutics, School of Pharmacy, Fourth Military Medical University, Xi’an, China; ^5^Department of Pharmacy, Ninth Hospital of Xi’an, Xi’an, China

**Keywords:** gallic acid, pharmacokinetics, myocardial infarction, isoproterenol, HPLC

## Abstract

Gallic acid (GA) is a polyphenolic natural product widely distributed in food, beverage, and traditional Chinese herbs with beneficial effects on the cardiovascular system. In this research, a comparative study was conducted to investigate the possible difference of pharmacokinetic process in normal and isoproterenol-induced myocardial infarcted rats after oral administration of GA monohydrate with the dose of 50 and 100 mg/kg, respectively. Quantification of GA in rat plasma was achieved by using a simple and rapid high-performance liquid chromatographic method. The results revealed that pharmacokinetics of GA were greatly different between normal and pathological state. GA exhibited slower absorption into the bloodstream, and yielded 1.7-fold (50 mg/kg GA) and 1.3-fold (100 mg/kg GA) less values of area under concentration–time curve as well as 2.5-fold lower of maximum blood concentration (C_max_) in MI rats than those in normal rats. In addition, significant prolonged T_1/2_ and MRT as well as decreased CL were also registered in MI rats. Our findings suggest that myocardial infarction could alter the pharmacokinetic process of GA, and thus the potential pharmacokinetic differences of herbal preparations (or dietary nutrition) containing GA between normal and pathological conditions should be brought to the forefront seriously in clinical practice.

## Introduction

**FIGURE 1:**
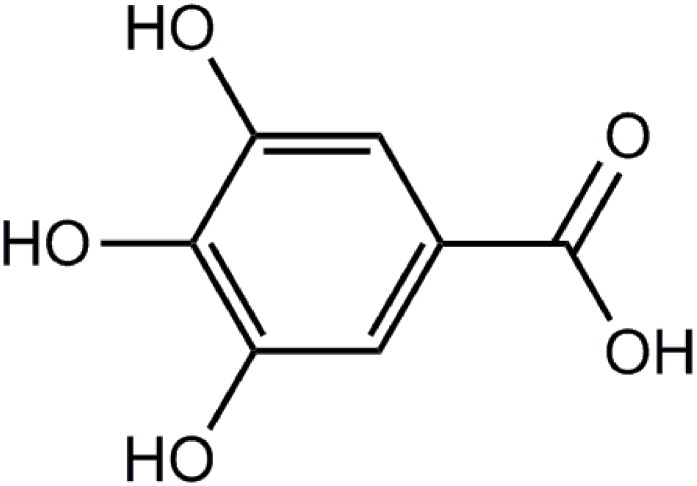
Chemical structures of gallic acid (GA) (chemical name: 3,4,5-trihydroxybenzoic acid).

Ischemic heart disease (IHD) is the most common cause of death in the world, taking 100s of millions of lives away over the decades. In recent years, the prevention of IHD has been associated with the ingestion of fresh fruits or herbs rich in natural antioxidants (Farías et al., 2017). The protective effects of plants can be due to the presence of flavonoids, anthocyanins, and phenolic compounds. Gallic acid (GA) (3,4,5-trihydroxybenzoic acid, **Figure 1**) is an endogenous plant polyphenol product (phenolic acid) which richly distributed in at least 12 plant species including *Rheum palmatum* L., *Rosa chinensis* Jacq., and *Paeonia suffruticosa* Andr. (Kosuru et al., 2017).

Also, as the active phenolic acid compound of many traditional Chinese herbs and herbal preparations, e.g., *Cortex Moutan* and *ShuangDan* Oral Liqued (Li et al., 2011), GA is reported to have memorably cardioprotective activity and receiving considerable attention in medical and nutritional research. A previous study has revealed that GA has beneficial effect on lysosomal enzymes, lipid peroxidation, and reduced glutathione in isoproterenol induced cardiac damage in rats (Stanely et al., 2009). Another report showed that GA could protect the heart by directly combination with free radicals and lead to inactivate them, which may suppress the intracellular concentration of free radicals (Priscilla and Prince, 2009). In addition, GA was proved to present negative chronotropic and inotropic effects in isoproterenol induced myocardial damage, which is similar to propranolol (Nwokocha et al., 2017).

Consistently with the extensive research into the cardioprotective activities of GA, a few reports had addressed the disposition profiles of GA *in vivo*. C8-modified graphene@mSiO2 composites based method and UPLC–MS/MS method have been applied to the kinetic studies of GA (Ma et al., 2015; Xu et al., 2017; Zhu et al., 2017). However, most of these work were carried out with the administration of crude drugs and/or Chinese patented medicines which content GA. Only in a few work, e.g., the previous studies of Shahrzad and Bitsch (1998), Yasuda et al. (2000), and Shahrzad et al. (2001), metabolic and primary pharmacokinetic properties of GA were studied with high-performance liquid chromatography (HPLC) methods after oral administration of GA monomer. Further, little work has been carried out to investigate the effects of ischemic condition on pharmacokinetics of GA and their related mechanisms. As we all know, remedies are used to treat diseases and in most cases patients are the ultimate consumers of them. It is possible that drug metabolic enzymes, transporters, cell membrane permeability and the change of microbe group could be affected by physiological and pathological changes, which enable the pharmacokinetics of GA in the body to be altered, including the process of absorption, distribution, metabolism, and excretion.

Therefore, the aim of our study was to investigate the possible difference of pharmacokinetic process in normal and isoproterenol-induced myocardial infarcted rats after oral administration of GA monohydrate. Pharmacokinetic data could provide additional information on preclinical and clinical use of herbal preparations as well as dietary nutrition comprising GA.

## Materials and Methods

### Materials and Reagents

Gallic acid monohydrate (99.0%, purity) was supplied by Jin Lv Biotech. Co., Ltd. (Xi’an, China); reference compound of GA (99.0%, purity) was purchased from the National Institute for the Control of Pharmaceutical and Biological Products (Beijing, China); 4-acetamidophenol (99.0%, purity) used as internal standard (IS) was purchased from the SinoPharm Chemical Reagent, Co., Ltd. (Shanghai, China). Isoproterenol hydrochloride was purchased from Tokyo Chemical Industry, Co., Ltd. (Kita-Ku, Tokyo, Japan). Methanol (HPLC grade) was obtained from Duksan Pure Chemicals (Ansan, South Korea). Glacial acetic acid (HPLC grade) was obtained from Mallinckrodt Baker (Phillipsburg, NJ, United States). The deionized water was prepared from Millipore water purification system (Milford, MA, United States) and was filtered with a 0.22-μm membrane. Other biochemical reagents and chemicals were all of analytical grade.

### Animals

Male Sprague-Dawley rats were purchased from Experimental Animal Research Center, the Fourth Military Medical University (Xi’an, China). They were kept in the individually ventilated cages (IVCs) system under controlled experimental conditions of 12 h-light/dark cycle for at least 5 days before starting the experiments and fed with standard laboratory food and water *ad libitum*. The rats (320∼350 g) were fasted (except for water) for 12 h prior to the experiment of oral administration of GA. Animal experiments were carried out in accordance with the policies and guidelines of Institutional Animal Care and Use Committee (IACUC) of the Fourth Military Medical University. The experimental protocols involving animals were reviewed and approved by the IACUC of the Fourth Military Medical University (Ethical Clearance No. KY2016-3034-1). The animal welfare, care, and use were managed by professionally well-trained technical personnel with relative educational background. All efforts were made to minimize animal suffering and to reduce the number of animals used.

### Induction of Myocardial Injury

Myocardial injury was induced in model group by subcutaneous (s.c.) injection of 85 mg kg^–1^ of ISO daily for 2 consecutive days to rats (Li et al., 2012, 2016). After 12 h of last dose of ISO administration, rats were anesthetized with pentobarbital sodium (35 mg kg^–1^, i.p.), blood was collected by abdominal aorta and allowed to clot for 1 h at room temperature. Serum was subsequently separated by centrifugation at 4000 rpm for 15 min and stored at –80°C for biochemical assays. After blood collection, rats were sacrificed by cervical decapitation. Heart and small intestine tissue was excised and rinsed immediately in ice-cold normal saline, then frozen in liquid nitrogen and stored at –80°C for further analysis.

### Assay of Serum Myocardial Injury Markers

Activity of serum creatine kinase-MB (CK-MB) was measured using commercial kit (Biosino Bio-technology and Science, Inc., Beijing, China). Serum levels of cardiac troponin I (cTnI) was estimated using standard commercial kits (Jiancheng Bio-technology and Science, Inc., Nanjing, China).

### Triphenyl Tetrazolium Chloride (TTC) and Hematoxylin and Eosin (H&E) Staining

Direct TTC assay according to our previous work (Li et al., 2012) was used to determine myocardial infarct size. In brief, the heart was transversely cut across both ventricles and sectioned into 2 mm thick. Sections were incubated at 37°C in 1% TTC-phosphate buffer (pH 7.4) for 30 min, following which they were fixed with 4% paraformaldehyde for 24 h. The non-ischemic myocardium and viable ischemic myocardium were stained red, while the infarcted myocardium appeared pale gray or white. The slices were photographed using a digital camera, and the % infarction was analyzed using the computerized Image-Pro Plus 6.0 software (Media Cybernetics, Inc., Silver Spring, MD, United States).

The tissues from ecardiac apex were fixed with 4% buffered paraformaldehyde for paraffin embedding. Sections were cut (by Leica RM2016 Microtome) into 5 μm and stained with hematoxylin and eosin (H&E). The sections were examined under light microscope (Nikon Eclipse E100) and micro graphed by Nikon DS-U3 digital camera controller at 200× magnification.

### Drug Analysis

Plasma concentrations of GA were determined by HPLC-UV method which has been firstly developed and validated. Briefly, HPLC analysis was performed on a Woburn C_18_ column (250 mm × 4.6 mm, 5 μm, Woburn) using LC2010-AT chromatographic system (Shimadzu, Kyoto, Japan). The mobile phase consisted of methano-2% glacial acetic acid solution (5:95, v/v) at a flow rate of 1 mL min^–1^. The temperature of column was maintained at 30°C. The detection wavelength was set at 272 nm. 4-acetamidophenol was used as IS.

To 200 μL plasma, 50 μL of 4-acetamidophenol (40 μg mL^–1^), 100 μL of 1 mol/L hydrochloric acid as well as 1 mL ethyl acetate were added and vortexed for 3 min. Followed by centrifugation at 4000 rpm for 15 min, 1 mL aliquot of the supernatant was transferred and the residue was mixed with 1 mL of ethyl acetate for another same extraction procedure. Finally, both of the supernatants were combined and evaporated to dryness at 40°C under a gentle stream of nitrogen. The dried residue was then reconstituted in 200 μL of mobile phase. After being vortexed for 1 min, the content was centrifugated at 12000 rpm for 15 min. The supernatant was then transferred to 2 mL glass vials and an aliquot of 20 μL was injected for analysis.

The calibration curve for GA was linear over the concentration range of 0.025–5 μg mL^–1^ with correlation coefficient (R) greater than 0.9999. The lower limit of quantification was 0.025 μg mL^–1^ (S/N ≥ 10). Quality control samples were prepared at 0.078, 0.63, and 2.50 μg mL^–1^. The relative standard deviation (RSD) of intra-day and inter-day precisions was less than 1.09% (*n* = 5). The relative errors (REs) for assay accuracies were found to be within 0.98 to 2.60%. The mean extraction recoveries at three quality control levels were between 87.31 and 90.02% (*n* = 5).

### Pharmacokinetic Study

For pharmacokinetic study, the rats were randomly and equally divided into four groups including two normal group and two myocardial infarction (MI) group (*n* = 8 for each group). Normal group was treated by oral administration of GA at doses of 50 and 100 mg/kg, respectively. MI group was inducted with ISO for myocardial injury and were treated by oral administration of GA at 50 and 100 mg/kg, respectively. Dose of GA were selected on the basis of previous studies (Hsu and Yen, 2007; Yousuf and Vellaichamy, 2015; Oyagbemi et al., 2016) and our pilot study. Approximately 300 μL (400 μL for the upper limit value) (NIH, 2015) blood samples were collected via retro-orbital sinus and transferred into heparinzed tubes at 0 (prior to dosing, serve as control), 10, 30, 60, 90, 120, 180, 300, 420, 540, and 600 min after oral administration. The technical personnel involved were well-trained in blood draws via retro-orbital sinus such that it imparts minimal pain to the animals. The rats were free access to 0.9% saline after collection time point (or administered equal volume of 0.9% saline orally if necessary) for fluid replacement. 200 μL of plasma was harvested by centrifuging the blood sample at 4°C and 4000 rpm for 15 min, and then stored at –80°C until analysis.

### Western Blot Analysis

Expressions of tight junction (TJ) proteins in small intestine of rats were analyzed as previously described immunoblotting method (Li et al., 2016) using the following antibodies: claudin-1, occluding, zo-1, and β-actin (Cell Signaling Technology, Danvers, MA, United States). Immunoreactive proteins were visualized by enhanced chemiluminescence using ECL kit (Guge Biotechnology, Wuhan, China). The signal intensity was detected and quantified by ChemiScope 5300 Pro System (Clinx Science Instruments, Co., Ltd., Shanghai, China).

### Statistical Analysis

The pharmacokinetic parameters of GA were analyzed through non-compartmental analysis using Drug and Statistics version 2.0 (DAS 2.0, Anhui Provincial Center for Drug Clinical Evaluation) software, including the half-life (T_1/2_), mean residence time (MRT), area under the plasma concentration-time curve (AUC), clearance (CL/F), and volume of distribution (V_d_/F). Data were shown as mean ± standard deviation (mean ± SD). The difference between groups was analyzed with the Student’s *t*-test using GraphPad Prism 6.0 statistical package program (GraphPad Software, Inc., La Jolla, CA, United States). *p*-Value less than 0.05 was considered as statistically significant.

## Results and Discussion

Gallic acid is an endogenous polyphenol found abundantly in medicinal plant. In recent years, some pharmacokinetic studies on crude drugs and/or Chinese patented medicines which content GA has been performed on healthy volunteers (Shahrzad and Bitsch, 1998) and normal animals (Xu et al., 2017; Zhu et al., 2017). However, considerable uncertainty remains due to the lack of comprehensive kinetic profiles after administration of GA monomer *in vivo*, especially under pathological condition. In the present study, the pharmacokinetic characteristics of GA were investigated in detail for the first time, and they were found to be great difference in normal and ISO-induced myocardial infarcted rats.

**FIGURE 2:**
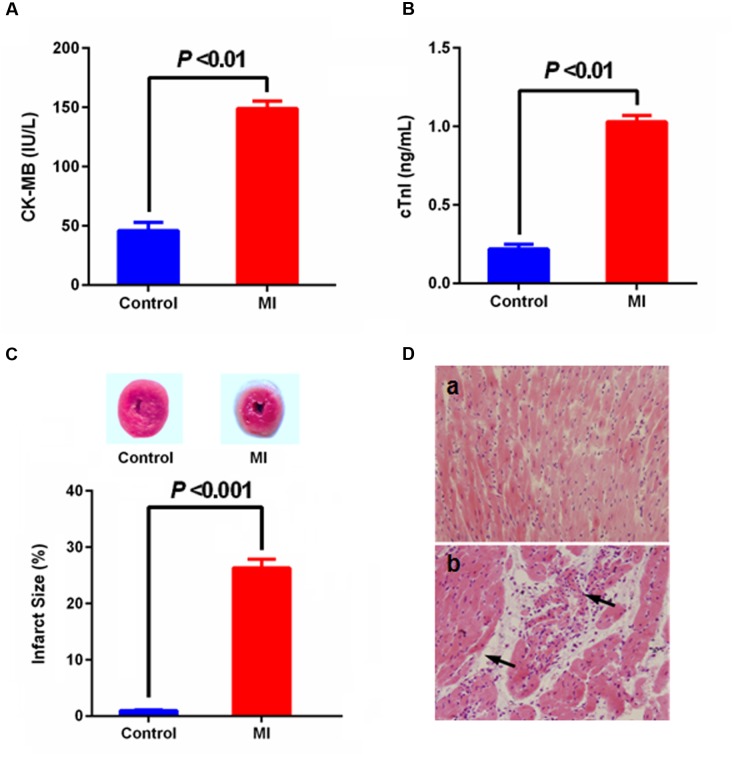
Effect of myocardial infarction (MI) on serum-specific cardiac biomarkers [**(A)** CK-MB, **(B)** cTnI; values are expressed as mean ± SD (*n* = 8)], **(C)** myocardial infarct size [normal myocardium was stained red, while pale gray areas indicate infarct areas; values are expressed as mean ± SD (*n* = 4)] and histopathologic changes of heart [**(D)** (a) control, (b) MI; heart tissues were stained with hematoxylin and eosin and visualized under light microscope at 200× magnification].

Myocardial infarction is a common presentation of IHD. It is the acute condition arising from the critical imbalance between coronary oxygen supply and myocardial demand, resulting in necrosis of the myocardium. ISO in supramaximal doses induces morphological and functional alterations in the heart leading to subendocardial myocardial ischemia, hypoxia and necrosis, which closely resembles local MI-like pathological changes seen in human MI (Li et al., 2016). During MI condition, cardic CK-MB and cardiac troponin I (cTnI) were released from the damaged heart tissue into the blood stream. Thus, levels of these specific cardiac biomarkers serve as sensitive indices to assess the severity of MI. As shown in **Figure 2**, there was a significant (*p* < 0.001) rise observed in the levels of CK-MB and cTnI the serum of group ISO-administered rats in comparison with normal rats, and abnormal histoarchitecture (e.g., loss of myofibrillar alignment and infiltration of inflammatory cells) of heart tissues were also observed in the ISO-induced rats, indicating the leakage and loss of functional integrity and/or necrotic damage of cell membrane. These results were in accordance with the direct staining of myocardial tissue using TTC dye, which forms a red formazan precipitate with the intact dehydrogenase enzyme systems of the viable myocardial tissue, whereas the infracted myocardium lack dehydrogenase activities thus fails to stain with TTC. ISO-induced rats showed increase in myocardial infarct size with less TTC absorbing capacity, indicating that the myocardial injury model was successfully induced thus can largely imitate the pathological condition of MI.

**FIGURE 3:**
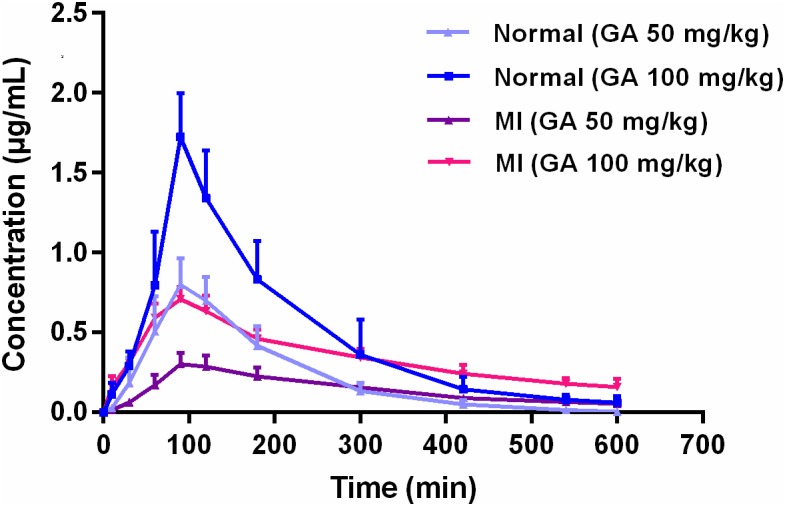
Mean plasma concentration-time profiles of gallic acid in normal and MI rats after oral administration of 50 and 100 mg/kg GA, respectively.

**FIGURE 4:**
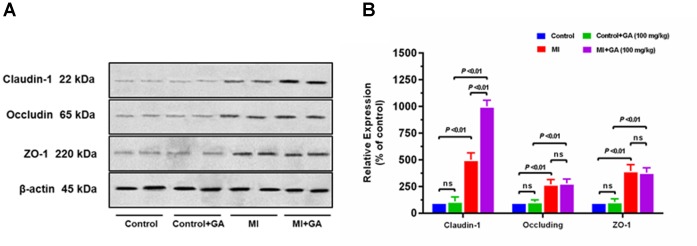
Effect of myocardial infarction and GA (100 mg/kg) on expressions of tight junction proteins in small intestine. Graphs represent **(A)** immunoblot of small intestine claudin-1, occluding, and zo-1 protein expressions and densitometry analysis of **(B)** claudin-1, occluding, and zo-1 protein expressions. Tissue samples were harvested after the last time point of blood collection. Values are expressed as mean ± SD (*n* = 4); ns, not significant.

Pharmacokinetics of GA in rat plasma after dosage of oral GA monohydrate of 50 and 100 mg/kg between normal and ISO-induced myocardial infarcted rats was then compared. The mean plasma concentration-time profiles of GA in normal and MI rats were presented in **Figure 3**. The main pharmacokinetic parameters of GA were calculated by the non-compartmental model and results were summarized in **Table 1**. After oral administration, the T_max_ for intact GA in normal rats (90 min for 50 mg/kg GA and 85 min for 100 mg/kg GA, respectively) and ISO-induced myocardial infarcted rats (102 min for 50 mg/kg GA and 96 min for 100 mg/kg GA, respectively) had no significant change, although ISO treated rats showed a slight delay. There were no significant differences in the T_1/2_ and V_d_ values when the GA dose was increased from 50 to 100 mg/kg body weight, while it was worth noting that the C_max_ and AUC of GA increased with dose in a dose-proportional manner at the doses of 50 and 100 mg/kg in both ISO groups and normal group, indicating that the pharmacokinetic behavior of GA could be in a dose-dependent manner. Moreover, it was well to be reminded that MI could significantly decrease the C_max_, CL, and AUC values whereas increase the T_1/2,_ MRT, and V_d_ values. C_max_ values of GA were estimated to be 0.32 μg mL^–1^ (50 mg/kg GA) and 0.72 μg mL^–1^ (100 mg/kg GA) in MI rats, which were approximately 2.5-fold lower than those in the respective dosed normal rats. The AUC_0–t_ of MI rats was also found to be decreased 1.7-fold (50 mg/kg GA) and 1.3-fold (100 mg/kg GA) when compared to respective dosed normal rats. Konishi et al. (2003) have reported that the permeation of GA was not in a polarized manner and was independent of pH, which indicate that the transepithelial transport of GA is permeated via the paracellular diffusion. Interestingly, results of our immunoblotting analysis (**Figure 4**) showed that MI condition significantly increased the expression of claudin-1, occludin, and zo-1 proteins in the small intestine when compared to normal rats. Claudin-1, occluding and zo-1 belong to the family of proteins that are the most important components of the TJs, where they establish the paracellular barrier that controls the flow of hydrophilic small molecules in the intercellular space between intestinal epithelial cells (Suzuki et al., 2017). Thus, we hypothesized that the poorer permeability through the intestinal epithelial membrane is more likely to be responsible for the decreased values of T_max_, AUC_0–t_, and C_max_ of GA after MI. Furthermore, significant prolonged T_1/2_ and MRT as well as decreased CL were also registered between normal and MI rats. It has been reported that when MI occurred, the volume of blood plasma was often decreased and drug clearance may also be diminished due to decreased cardiac output and blood flow to the major organs such as liver and kidneys, as well as decreased hepatic drug metabolizing activity (Imanparast et al., 2017). Li et al. (2015) found that MI rats induced by pituitrin had a longer half-life time and MRT of notoginsenoside compared with the normal rats, indicating pathological state of MI might cause variation of absorption and metabolism after drug was orally administered compared with those under the normal state. In addition, the elimination rate of GA slowed down while AUC_0–∞_ showed a slight increase in MI rats, which suggest the drug might cumulate in the pathological state. Therefore, it is necessary to investigate the dynamics of GA to enhance its safety and efficacy during clinical applications.

**Table 1:** Pharmacokinetic parameters of GA in normal and ISO-induced myocardial infarcted rats after oral administration of 50 and 100 mg/kg GA, respectively (mean ± SD, *n* = 8).

Pharmacokinetic parameters	Units	GA (50 mg/kg)		GA (100 mg/kg)	
		Normal	MI	Normal	MI
C_max_	μg⋅mL^–1^	0.83 ± 0.15	0.32 ± 0.11*	1.71 ± 0.29	0.72 ± 0.19*
T_max_	min	90.00 ± 21.21	102.00 ± 15.49	85.00 ± 12.25	96.00 ± 13.42
T_1/2_	min	153.65 ± 19.45	244.51 ± 26.23*	186.67 ± 23.97	278.17 ± 26.86*
AUC_0–t_	mg⋅min⋅L^–1^	137.07 ± 24.75	83.41 ± 18.17*	262.55 ± 22.05	198.99 ± 24.69*
AUC_0–∞_	mg⋅min⋅L^–1^	150.49 ± 26.35	121.62 ± 17.16	280.49 ± 26.35	300.52 ± 37.33
MRT_0–t_	min	160.08 ± 18.58	238.58 ± 14.89*	176.64 ± 24.03	274.77 ± 7.29*
MRT_0–∞_	min	187.97 ± 21.75	301.21 ± 42.29*	211.58 ± 24.54	331.21 ± 43.89*
CL	L⋅(min⋅kg)^–1^	0.37 ± 0.08	0.29 ± 0.10	0.43 ± 0.06	0.34 ± 0.04*
V_d_	L⋅kg^–1^	78.52 ± 8.51	131.09 ± 24.16*	89.60 ± 10.53	113.09 ± 13.58*

*^∗^p < 0.05 compared with respective normal rats.*

## Conclusion

In summary, our findings suggest that the pharmacokinetic process of GA could be altered in the condition of myocardial ischemia, and the parameters obtained from the normal state are limited and needs to be modified according to the practice. Kinetic features of GA obtained from the present study could be applied as a reference for evaluating its clinical efficacy.

However, it should be mentioned that in the present study approximately 1.5% of total circulating blood volume (CBV) was taken per time point/rat, which was greater than the volume (1% CBV) recommended by NIH (NIH, 2015). Reports have addressed that pharmacokinetic behavior of some drugs could be altered by supramaximal fluid loss (Kugelberg et al., 2006), though some could not (Sue et al., 1989). Whether the lager amount of blood sampling has potential effects (or bias) on the pharmacokinetic behavior of GA is unclear. Studies will be conducted to make this issue clear in the further work. In addition, it should also be note the effects of many drugs could be attributed partially to the action of their metabolites, rather than the direct action of the parent drug (Yasuda et al., 2000). Thus, further investigations are necessary to focus on the absorption mechanism of both GA and its metabolites in gastrointestinal track between normal and pathological conditions.

## Author Contributions

ZY and HL conceived and designed the experiments, performed the experiments, and wrote the original manuscript. FS performed the experiments and analyzed the data. Y-CJ and W-MZ analyzed the data, prepared figures, and crafted the final manuscript. YZ and E-JL helped with the experiments and prepared figures. DZ, L-LB, and QY helped with the analysis of data. HL collected data, provided constructive comments, and crafted the manuscript. B-LZ and S-WW conceived and supervised the study. All authors reviewed and approved the final manuscript.

## Conflict of Interest Statement

The authors declare that the research was conducted in the absence of any commercial or financial relationships that could be construed as a potential conflict of interest.
